# Supplementary material for: A genome-wide copper-sensitized screen identifies novel regulators of mitochondrial cytochrome c oxidase activity
Source: J Biol Chem. 2021 Mar 1;296:100485. doi: 10.1016/j.jbc.2021.100485 (PMC8027276; doi:10.1016/j.jbc.2021.100485)
Supplement: Figures — S1 to S4 [file mmc1.pdf]

Supporting Information

Title: A genome wide copper-sensitized screen identifies novel regulators of mitochondrial cytochrome *c* oxidase activity

Natalie M. Garza, Aaron T. Griffin, Mohammad Zulkifli, Chenxi Qiu, Craig D. Kaplan, Vishal M. Gohil*

Table of Contents

Supplementary Figure 1.....S-2
Supplementary Figure 2.....S-3
Supplementary Figure 3.....S-4
Supplementary Figure 4.....S-5
Description of Supporting Data Not Provided in PDF Format.....S-6

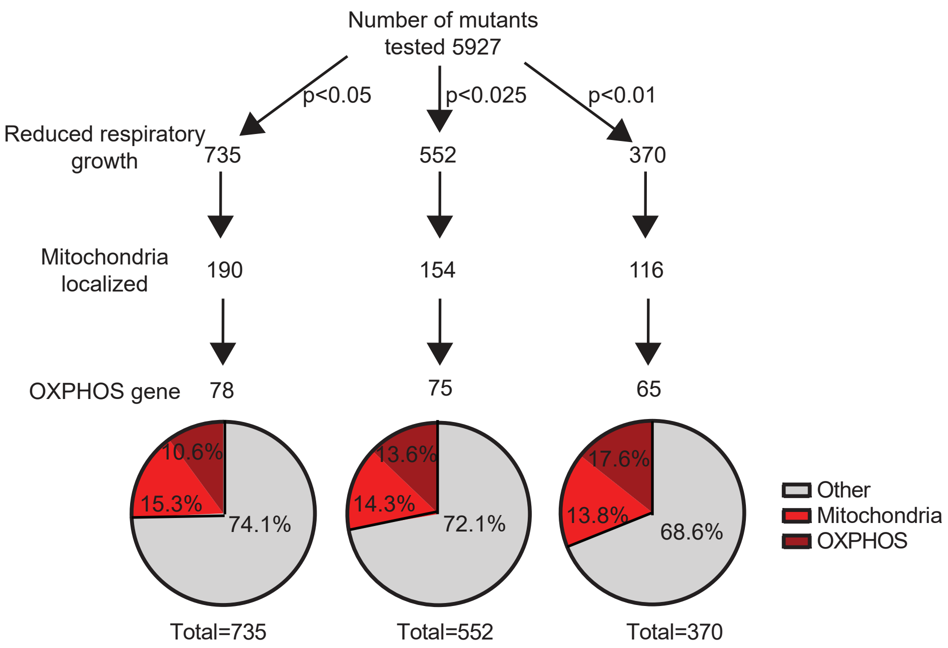

Supplementary Figure S1. Enrichment of OXPHOS and other mitochondrial genes increases with increased stringency of p-value threshold.
Illustration of the number of respiratory deficient strains identified at each p-value threshold. The distribution of hits between known OXPHOS protein encoding genes (maroon), other mitochondrial protein encoding genes (red), and non-mitochondrial protein encoding genes (grey) is shown.

Other genes encoding mitochondrial proteins

TCA Cycle
Fum1
Idh1
Kgd1
Kgd2
Mdh1

Mitochondrial DNA expression
Aim23
Mba1
Mod5
Mrp49
Mrpl1
Mrpl39
Mrx6
Msc6
Oms1
Pet20
Ppe1
Pth2
Pth4
Qri7

Additional mitochondrial hits
Aat1
Aim17
Aim24
Aim26
Ald4
Apj1
Cym1
Ddl1
Gep7
Gut2
Iai11
Mcp2
Mfm1
Mgr1
Mic60
Mrs2
Nfu1
Num1
Pis1
Puf3
Put1
Rrg7
Sap185
Sod2
Tim21
Uth1
Yhm2
Ysc83

Glutathione biosynthesis
Gsh1
Gsh2

Rim101 pathway
Dfg16
Rim8
Rim9
Rim13
Rim20
Rim21
Rim101

AP-3 Complex
Apl5
Apl6
Apm3
Aps3

Other vesicle mediated transport
Age2
Apl2
Apl4
Apm1
Aps1
Arf2
Arl3
Avl9
Bmh2
Ccz1
Did4
Gcs1
Get1
Get2
Gyp1
Inp53
Irc6
Mon1
Pep8
Ras2
Ric1
Rtt10
Rvs167
Shr3
Snf8
Svp26
Swa2
Sys1
Tda3
Vab2
Vam7
Vam10
Vps5
Vps17
Vps29

Other vesicle mediated transport continued
Vps35
Vps41
Vps51
Vps63
Yck3
Yip4
Ypt7

Ion and other transport
Ant1
Atx1
Avt5
Ccc1
Erc1
Flc1
Flc2
Flc3
Fre7
Ftr1
Gdt1
Gef1
Hxt8
Kha1
Mch5
Nhx1
Osh3
Rav1
Rav2
Sam3
Stl1
Stv1
Vtc5
YDL199C
YGR125W
YOL162W

Other hits
Aht1
Adh6
Apt2
Arg80
Asi3
Azf1
Ber1
Bmh1
Bye1
Car1
Car2
Cat8
Cnn1
Cti6
Ctt1
Cub1
Cys4
Dat1
Dbr1
Dcs1
Ddr48
Eaf7
Edc3
Erv2
Far10
Fbp1
Fbp26
Fsh1
Fyv8
Gal11
Gal7
Gal80
Gal83
Gdh1
Glc8
Gzf3
Hap4
Hap5
Hda2
Hda3
Hel2
Hir1
Hir2
Irc14
Isw2
Jnm1
Kns1
Ktr2
Ldb18
Lug1
Mak10
Mbr1
Mdh2
Mds3
Met16
Mft1
Mnl2
Mrk1
Mth1
Msc6
Msn1
Nab6
Nbp2
Ndl1
Ngl2
Nis1
Npr1
Npr2
Nsi1
Ntg2
Nto1
Nut1
Opi1
Orm2
Par32
Pcl6
Pcl8
Pho2
Pho4
Pph21
Ppm1
Ppq1
Psp2
Psy2
Ptk1
Ptp3
Pub1
Pus1
Pyc1
Rck2
Rim11
Rmd1
Rmd8
Rot2
Rpl16B
Rrd2
Rtc1
Rvs167
Sas3
Scs22
Sds3
Ser3
Sgn1
Ski7
Sko1
Smi1
Snf3
Snf4
Sok1
Sok2
Spg3
Spo7
Ssa1
Ste20
Syh1
Toh1
Tor1
Trm10
Ubr1
Ume1
Ura4
Vba4
Vhs1
Vms1
Vps61
YAL004W
Yap5
YBL044W
YBL055C
YBL096C
YBR224W
YBR225W
YBR226C
YDL068W
YDL172C
YDR048C
YDR269C
YDR271C
YDR391C
YDR455C
YER097W
YGL199C
YGL235W
Ygp1
YHL005C
YJR087W
YJR120W
YKL136W
YLL020C
YLR169W
YLR202C
YLR269C
YLR345W
YML082W
YML089C
YML090W
YMR057C
YMR315W
Yng1
Ynk1
YNL140C
YNL179C
YNR042W
YOL036W
YOR139C
YOR333C
YPL150W
YPL182C
YPR012W
YPR039W
YPR050C
YPR114W
YPR123C
Yps7
Yta7

Supplementary Figure S2. Respiratory deficient mutants of non-OXPHOS related proteins. Non-OXPHOS associated proteins with a p-value ≤0.01 for T-score (YPGE-YPD) were grouped into different functional categories.

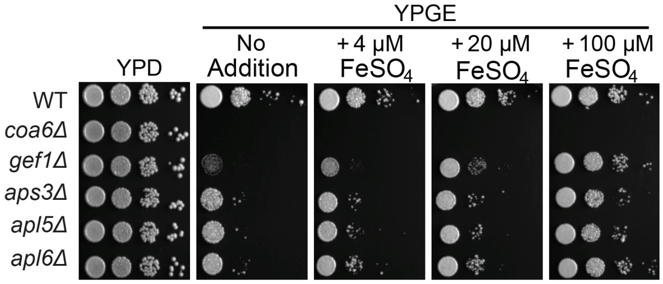

Supplemental Figure S3. Respiratory growth of AP-3 mutants is restored by high iron supplementation. Serial dilutions of WT and the indicated mutants were seeded onto YPD and YPGE plates with and without 4, 20 or 100 μM $FeSO_4$ at 37°C and grown for two (YPD) or four days (YPGE). Here *gef1Δ* cells were used as a positive control for iron-based rescue.

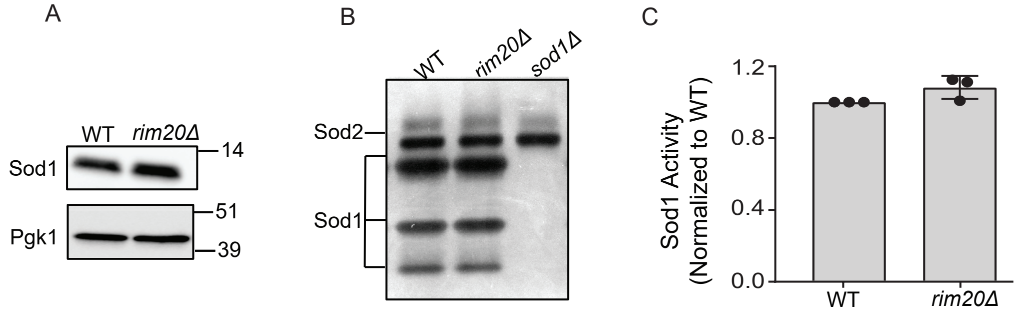

Supplementary Figure S4. Activity of cytosolic cuproenzyme Sod1 is not altered in *rim20Δ* cells. (A) Crude cytosolic fraction was analyzed by SDSPAGE/western blot to detect Sod1 abundance in the indicated strains. Pgk1 was used as a loading control. (B) Sod activity in isolated crude cytosolic fraction from the indicated strains was measured by in-gel assay as described in Methods. (C) For quantification of the Sod1 activity in (B), the images were digitalized and densitometry analysis was performed using the Image Lab software.

Description of Supporting Data Not Provided in PDF Format

Table S1: Excel file containing a rank ordered list of mutants' growth in YPGE compared to YPD.
Table S2: Excel file containing a list of yeast genes encoding OXPHOS proteins and assembly factors.
Table S3: Excel file containing a rank ordered list of mutants' growth in YPGE+Cu compared to YPGE.
